# Daily Artificial Gravity Partially Mitigates Vestibular Processing Changes Associated with Head-down Tilt Bedrest

**DOI:** 10.21203/rs.3.rs-3157785/v1

**Published:** 2023-07-19

**Authors:** Rachael Seidler, Grant tays, Kathleen Hupfeld, Heather McGregor, Nichole Beltran, Yiri de Dios, Edwin Mulder, Jacob Bloomberg, Ajitkumar Mulavara, Scott Wood

**Affiliations:** University of Florida; University of Florida; University of Florida; University of Florida; KBR; KBR; German Aerospace Centre (DLR); NASA Johnson Space Center; KBRwyle

## Abstract

Microgravity alters vestibular signaling and reduces body loading, driving sensory reweighting and adaptation. The unloading effects can be modelled using head down tilt bedrest (HDT). Artificial gravity (AG) has been hypothesized to serve as an integrated countermeasure for the physiological declines associated with HDT and spaceflight. Here, we examined the efficacy of 30 minutes of daily AG to counteract brain and behavior changes that arise from 60 days of HDT. One group of participants received 30 minutes of AG daily (AG; n = 16) while in HDT, and another group served as controls, spending 60 days in HDT bedrest with no AG (CTRL; n = 8). We examined how HDT and AG affect vestibular processing by collecting fMRI scans from participants as they received vestibular stimulation. We collected these data prior to, during (2x), and post HDT. We assessed brain activation initially in 10 regions of interest (ROIs) and then conducted an exploratory whole brain analysis. The AG group showed no changes in brain activation during vestibular stimulation in a cerebellar ROI, whereas the CTRL group showed decreased cerebellar activation specific to the HDT phase. Additionally, those that received AG and showed little pre- to post-bed rest changes in left OP2 activation during HDT had better post-HDT balance performance. Exploratory whole brain analyses identified increased pre- to during-HDT activation in the CTRL group in the right precentral gyrus and the right inferior frontal gyrus specific to HDT, where the AG group maintained pre-HDT activation levels. Together, these results indicate that AG could mitigate brain activation changes in vestibular processing in a manner that is associated with better balance performance after HDT.

## 1. Introduction

Following spaceflight, astronauts return to Earth with transient deficits in their balance and mobility; these can last for several weeks before performance returns to preflight levels (Tays et al., 2021; Mulavara et al., 2010). This is thought to be largely due to altered vestibular signaling and multisensory re-weighting that occur in microgravity. Vestibular inputs in a microgravity environment, particularly from the otoliths (which detect linear accelerations and head tilt relative to gravity) are down-weighted because the signals are unreliable in the absence of gravity (Hupfeld et al., 2022; Boyle, 2021). Once an astronaut returns to Earth, adaptive responses become maladaptive in the presence of gravity, temporarily hindering locomotion and balance performance (Mulavara et al., 2018). Studies have also shown vestibular neural changes either during flight or post-flight, such as reduced in-flight electroencephalography alpha power localized to the vestibular, motor and cerebellar brain regions (Cheron et al., 2006; 2014; Cebolla et al., 2016). Additionally, a case study reported decreased vestibular resting state network connectivity postflight (Demertzi et al., 2016). Our own recent work has supported the sensory re-weighting view, based on analyzed functional magnetic resonance imaging while astronauts received vestibular stimulation pre- and post-flight. Typically, vestibular stimulation elicits brain deactivation in cross modal sensory regions (Noohi et al., 2014; 2017; 2019). However, following ~ 6 months of spaceflight, among 15 astronauts, we identified widespread pre- to post-flight reductions in this brain deactivation across sensorimotor, frontal, temporal and occipital regions; we interpreted this increased deactivation as suggestive of upweighting of somatosensory and visual processing during flight (when vestibular inputs are unreliable) (Hupfeld et al., 2022). Further, we identified a brain-behavior correlation between pre- to post-flight activation change in visual and multisensory integration brain regions and pre- to post-flight change in balance. Those that had greater reductions in deactivation of these brain regions from pre- to post-flight maintained better balance performance (Hupfeld et al., 2022). In sum, spaceflight impacts the brain’s processing of vestibular stimuli and sensory weighting for motor control.

Long duration head-down tilt bedrest (HDT) has been repeatedly used as a spaceflight analog to model physiological changes that occur in microgravity. Participants lie with their head 6° below their feet, resulting in headward fluid shifts, axial body unloading and other physiological effects of microgravity (Hargens and Vico, 2016; Reschke et al., 2009; Mulder et al., 2014; Koppelmans et al., 2015, 2017; Miller et al., 2018; Mulavara et al., 2018; Tays et al., 2022). HDT does not directly affect vestibular inputs, however it is thought to initiate sensory re-weighting which will indirectly affect processing as the vestibular nuclei also receive proprioceptive inputs from the limbs (Rubin et al., 1979; Yates et al., 2000; Jian et al., 2002). During bedrest, subjects are deprived of higher frequencies of linear accelerations that are associated with locomotion (MacDougall and Moore, 2005). If vestibular or somatosensory inputs from the limbs are disrupted, the central nervous systems may upregulate other sensory systems to compensate and maintain performance (Bles et al., 1984; Horak and Hlavacka, 2001; Carriot et al., 2015). During HDT typical somatosensory inputs to the foot sole are removed and vestibular processing appears to be altered as vestibular cues are upweighted, or relied upon more heavily (Mulavara et al., 2018; Hupfeld et al., 2020). Performance of behaviors that depend upon the vestibular system and multisensory integration, such as functional mobility and postural stability, have been shown to decrease following HDT (Reschke et al., 2009, Mulder et al., 2014; Koppelmans et al., 2015; 2017; Miller et al., 2018; Mulavara et al 2018; Tays et al., 2022).

We have previously demonstrated that HDT affects the neural correlates underlying vestibular processing (Yuan et al., 2018), and further, HDT in conjunction with elevated CO_2_ (such as occurs in the enclosed environment of the International Space Station (ISS)) affects vestibular processing (Hupfeld et al., 2020). We found that HDT results in increased activation in portions of the insular, frontal, and parietal cortices during vestibular stimulation, suggesting that more brain resources may be required to process vestibular information during HDT (Yuan et al., 2018). When investigating the added effects of CO_2_ to HDT, we identified increased activation from to pre-HDT to post-HDT in the left inferior temporal gyrus, right superior occipital gyrus and brainstem in those that had increased levels of CO_2_ relative to HDT alone (Hupfeld et al., 2020). Further, greater *deactivation* in various regions was associated with sustained behavioral performance in mobility and balance tasks (Hupfeld et al., 2020). That is, participants that had greater deactivation pre-to-post HDT had the best behavioral performance from pre-to-post HDT. Overall, HDT has been shown to influence vestibularly mediated, multi-sensory behavior and brain activation patterns that underlie vestibular processing. These changes in neural processing have also been associated with the behavioral changes that can occur in HDT, supporting the argument that HDT causes multi-system re-weighting. Using this same method of measuring vestibular processing, we found a similar pattern of deactivation in astronauts following their return from ~ six-month missions to the ISS (Hupfeld et al., 2022). Here, greater deactivation in visual regions of the brain from pre- to post-spaceflight was also found to be associated with better balance performance post-flight. Thus, HDT and spaceflight have been shown to have similar effects on vestibular processing and reinforce that HDT is a suitable analog environment to investigate these changes and potential countermeasures for spaceflight.

Countermeasures for postflight physiological and functional changes have been under investigation for many years. Since many different systems are affected by spaceflight (muscle, bone, cardiovascular, neural, etc., c.f. Clement et al., 2020), the ideal countermeasure would be integrated to target many systems at once. Artificial gravity (AG) has been proposed to provide multisystem benefits (Hargens et al., 2013; Clement et al., 2015). In spaceflight, the otoliths cannot signal head tilt, there is bone and muscle loss, proprioceptive sensors receive reduced stimulation, and there is cardiovascular deconditioning (Boyle, 2021). On Earth, AG can be applied along the long axis of the body via centrifugation. The participant lies in a supine position and is spun to create 1G at their center of mass. This protocol has previously been investigated with participants undergoing 5 days of HDT and receiving two different AG exposure protocols. AG applied intermittently (in 6 bouts of 5 minutes) was shown to mitigate decreases in orthostatic tolerance due to HDT (Linnarsson et al., 2015). Further, we used the same AG protocol in conjunction with NASA and ESA (Artificial Gravity Bed Rest-European Space Agency; AGBRESA) for a 60-day campaign of HDT to investigate the counter-active effects of AG on sensorimotor and cognitive performance (Clément et al., 2022). We found that AG may serve as a countermeasure for balance and mobility deficits that occur with HDT, and further, during centrifugation participants were able to perform better on a cognitive task than controls who performed in bedrest (Tays et al., 2022). This was supported by other work, finding that intermittent AG partially mitigated the deterioration in sway path and velocity, as well as sway frequency power (De Martino et al., 2021). However, Clements et al. (2022) analyzed balance performance in the AGBRESA campaign as well, and identified no mitigation effects of AG on balance performance pre-to post-HDT. In the same campaign, we found that AG increases neural efficiency during sensorimotor adaptation tasks (Tays et al., 2023).

In the current study, we tested vestibular processing in the same manner as our previous HDT (Yuan et al., 2018; Hupfeld et al., 2020) and spaceflight studies (Hupfeld et al., 2022). Our primary aim was to examine if AG applied along the long axis of the body mitigates vestibular processing changes that occur with HDT. We hypothesized that control participants would show greater pre- to post-HDT changes in brain activity during vestibular stimulation compared with those receiving the AG intervention. Further, we predicted that individual differences in brain changes in would be correlated with decreases in balance and mobility from pre- to post-HDT.

## 2. Materials and Methods

### 2.1 Participants

Twenty-four individuals (8 F; all individuals: 33.3 ± 9.17 yrs, 174.6 ± 8.6 cm, 74.2 ± 10.0 kgs) were recruited to participate in this study. All participants were screened for tolerability of AG, according to the AG2 protocol (Linnarsson et al., 2015), prior to enrollment. Participants were also selected to be of similar age (24–55 years), sex and education range to astronauts. Exclusion criteria included cardiovascular disease history, medications and smoking for 6 months prior to entering the experiment. They provided written informed consent prior to participating; the protocol was approved by the University of Florida and NASA Institutional Review Boards as well as the regional medical association (Ärztekammer Nordrhein). This study was a piece of the larger joint investigation conducted by NASA, the European Space Association (ESA) and the German Aerospace Center (DLR) to identify if AG could serve as an integrated countermeasure to target spaceflight induced functional changes. The entirety of the study was conducted at the DLR’s :envihab facility in Cologne, Germany. As with larger studies, participants engaged in various other experiments during this time; here, we will only discuss those relevant to this investigation. Participants were also given free time throughout the day on a fixed schedule where they could read, operate a computer or do as they see fit while remaining in the HDT position. We have previously published cognitive and sensorimotor brain and behavior data that were collected as part of this larger campaign (Tays et al., 2022; 2023).

All participants experienced 60 days of HDT with their head 6° below their feet. Prior to and following the 60 days of HDT they performed baseline and recovery testing, respectively. During the two weeks prior to entering HDT, participants experienced AG twice (11 days and 4 days prior) and then were randomized into three groups. Two of the groups received centrifugally applied AG for 30 minutes daily, either applied in one continuous bout, or intermittently for 6 bouts that consisted of 5 minutes each with 3 minutes between each bout. The third group was a control group that received no AG; all three groups spent the same time period in HDT. We initially examined for statistical differences in brain activation between the two AG groups and found none; thus we pooled them together into a joint AG group. AG was applied via a long arm centrifuge at the :envihab facility where rotational speed was customized with each participant based on their center of mass (CoM) to generate 1G in the z axis at their CoM and around 2Gz at their feet. Max speed was reached following a ramp up/down that did not exceed 5° s^− 2^ to reduce negative tumbling sensations that can arise from vestibular stimulation in this manner. The participants were instructed to keep their body as still as they could, but they were not restrained to enforce this.

**Figure 1:**
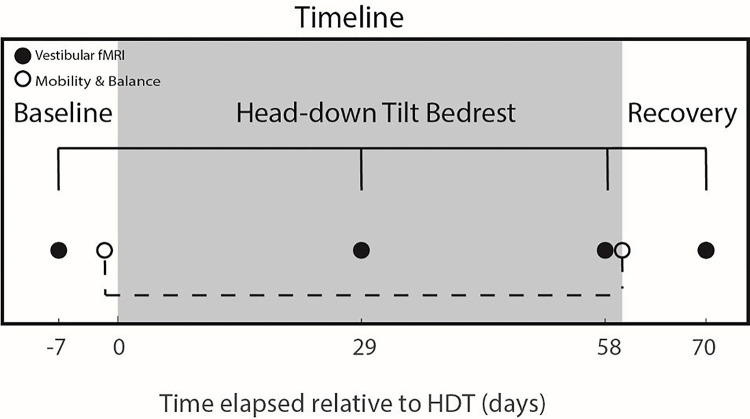
Task timeline. Functional MRI data were collected prior to HDT (7 days), twice during (29 and 58 days) and once following (10 days). Mobility and balance data for the brain-behavior correlation were collected one day prior to entering HDT and on the day participants exited HDT.

Here, we used a vestibular stimulation method that we have validated in healthy young adults (Noohi et al., 2017), and used in both HDT (Yuan et al., 2018; Hupfeld et al., 2020) and spaceflight studies (Hupfeld et al., 2022) while participants were in the MRI scanner. Vestibular stimulation was applied with a MRI compatible pneumatic tactile pulse system (Pn Tacticile Pulse System; PnTPS, Engineering Acoustics Inc.; cf. Wackym et al., 2012; Yuan et al., 2018; Noohi et al., 2017; 2019; Hupfeld et al., 2020; 2022). This tapper works using compressed air to deliver low force taps (0.6 kg) to the lateral cheekbones. This has been repeatedly shown to elicit vestibular-evoked myogenic potentials (measured with electromyography, EMG), and to activate vestibular cortical regions and deactivate somatosensory and visual cortices (measured with fMRI) (Noohi et al., 2017), and to be less irritating to participants than other vestibular stimulation methods (Wackym et al., 2012; Noohi et al., 2017; Brantberg et al., 2009). We measured brain activation during cheekbone taps compared with rest while participants were in the HDT position within an MRI scanner (described in further detail below). Participants underwent this stimulation four times (Fig. 1) for this experiment; 7 days pre-HDT (Baseline Data Collection (BDC) −7), 29 and 58 days in HDT (HDT29, HDT58) and 10 days post-HDT (recovery, R + 10).

### 2.2 Mobility and Balance Measures

We included multiple measures of mobility and balance that have been shown to change with both spaceflight and HDT. This allowed us to test for brain-behavior correlations, to better understand the functional relevance of any observed brain changes.

#### 2.2.1 Functional Mobility Test

The functional mobility test (FMT) is an obstacle course designed to require similar maneuvers as might be used to perform emergency egress from a space vehicle after landing (Mulavara et al., 2010). It requires participants to move as quickly as possible, while walking, through a 6m × 4m obstacle course, making maneuvers around various foam pylons, under foam hurdles and between slalom bars. The first half is on a solid surface, whereas the participants walk on high density foam (Sunmate Foam, Dynamic Systems Inc. Leicester, NC, USA) for the second half to perturb proprioceptive inputs. The FMT has been shown to be sensitive for detecting mobility changes that occur with spaceflight (Mulavara et al., 2010; Cohen et al., 2012; Mulavara et al., 2018; Tays et al., 2021), and HDT (Koppelmans et al., 2017; Lee et al., 2019; Reschke et al., 2009; Tays et al., 2022). The primary outcome measure is a participant’s completion time on the first trial of the obstacle course.

#### 2.2.2 Sensory Organization Tests

To assess balance, we utilized the Sensory Organization Test 5 and 5M (SOT-5; SOT-5M) conducted with a computerized dynamic posturography system (Equitest, NeuroCom International, Clackamas, OR). The SOT-5 required participants to remain in upright balance with their head upright, eyes closed and on a sway-referenced base. The SOT-5M consists of the same, however instead of maintaining their head in a rigid position they must perform dynamic head tilts of ± 20°, at 0.33 Hz rate given via a metronome tone (Wood et al. 2015).. Participants performed 3 trials, each 20s, and we used the median score. Equilibrium quotients (EQ) were calculated based on the peak-to-peak center of mass sway angle (θ), (EQ = 100 X [1−(θ/12.5°)]. The 12.5° represents the max theoretical limit of stability in the anterior-poster direction. Less sway during the task results in higher scores with a maximum of 100, interpreted as better performance. Note that both conditions are intended to assess one’s use of vestibular input for maintaining balance by depriving visual cues with eyes closed and disrupting proprioceptive feedback with the base of support that moves in proportion to body way.

### 2.3 fMRI Parameters

Vestibular stimulation was applied while fMRI data were acquired with a 3-Tesla Siemens Biograph MRI scanner located at the DLR’s :envihab facility. A gradient echo T2*-weighted echo-planar imaging sequence with the following parameters was used to acquire fMRI data: TR: 2500 ms, TE: 32 ms, flip angle: 90°, FOV: 192 × 192 mm, matrix: 64 × 64, slice thickness: 3.5 mm, voxel size: 3 × 3 × 3.5 mm, 37 slices. We also acquired a T1-weighted gradient-echo pulse sequence with the following parameters: TR: 1.9 s, TE: 2.4 ms, flip angle: 9°, FOV: 250 × 250 mm, matrix: 512 × 512, slice thickness: 1.0 mm, voxel size: 0.49 × 0.49 × 1.0 mm, 192 slices. The participants remained in the HDT position during the fMRI collection by lying on a foam wedge, however their head was flat within the head coil. Participants completed one fMRI run at each testing session. A run consisted of five, 24 second blocks of active tapping, with 20 second rest periods between each block. The taps were delivered at 1 Hz, with a total of 24 taps per block. Other fMRI tasks assessing visuomotor adaptation, dual-tasking and spatial work memory were conducted at the same time, but here we only focus on the vestibular fMRI.

### 2.4 Whole Brain fMRI Pre-processing

fMRI data were pre-processed using Statistical Parametric Mapping 12 (SPM12; version 7219; Penny et al., 2011), the Advanced Normalization Tools package (ANTs; Avants et al., 2011) and FSL command line tools (Jenkinson et al., 2012). This pre-processing pipeline is similar to what we have used in our past HDT work (Hupfeld et al., 2020; Salazar et al., 2020; 2021; Tays et al., 2023). Field maps were created to identify and correct B0 inhomogeneities with the FSL topup tool (Jenkinson et al., 2012). Then, images were corrected for slice timing, then realigned and resliced to correct for volume-to-volume head motion in SPM12. We used the Artifact Detection Tool (ART; https://www.nitrc.org/projects/artifact_detect/) to identify volumes with framewise displacement motion ≥ 2.0 mm and global brain signal Z threshold ≥ 9; all outlier volumes were then statistically covaried during analysis. To move each run into a standard MNI space, we used multivariate templates created with ANTs (Avants et al., 2011). First, for each participant we created a longitudinal T1 template across all time points with the AntsMultivariateTemplateConstruction.sh function. Next, using the same function we created participant specific fMRI multivariate templates with the fMRI data. These two templates were then co-registered using the AntsRegistration.sh function, and then the structural multivariate template was normalized to MNI space using the same function and an MNI152 template. The transformations created from these registrations were concatenated into a flow field and applied to the pre-processed fMRI images for each participant at each time point to bring their fMRI runs into standard space. Normalized images were then spatially smoothed with an 8mm full width at half-maximum three dimensional Gaussian kernel.

### 2.5 Cerebellar Pre-processing

As in our previous analog and spaceflight fMRI work (Salazar et al., 2020; 2022; Hupfeld et al., 2020; 2021; Tays et al., 2023), we used specialized pre-processing to remove and assess the cerebellum separately from the cerebral cortex. To do this, we used the CEREbellum Segmentation (CERES; Romero et al., 2017) pipeline and the Spatially Unbiased Infratentorial Template (SUIT; Diedrichsen, 2006; Diedrichsen et al., 2009). First, participant-specific T1 templates were uploaded to the CERES segmentation pipeline to isolate the cerebellum from the whole of the brain. The isolated cerebellum was then converted into a binary cerebellar mask from the CERES output using ImCalc in SPM12 and applied to each time point. Next, we used ANTs AntsRegistration.sh to transform the T1 cerebellar template into a standard space with the SUIT template. Then, each slice timed, realigned and resliced fMRI run was transformed to the participants T1 template space where it was masked with the individual’s cerebellar binary mask. Next, the masked fMRI data were transformed into SUIT template space using AntsApplyTransforms.sh. Finally, we applied a 2 mm full width at half-maximum three-dimensional smoothing Gaussian kernel to SUIT space cerebellar images in SPM12. We chose a 2 mm kernel here due to the small lobule size of the cerebellum, similar to other studies (Diedrichsen, 2006; Diedrichsen et al., 2009; 2011).

### 2.6 Mobility and Balance Behavioral Statistical Analyses

We used the nlme package (Pinheiro et al., 2020) in R 3.6.1 (R Core Team, 2019) to fit linear mixed effects models with restricted maximum likelihood (REML) estimation to examine mobility and balance performance changes over time. In each model we entered subject as a random intercept to allow for different starting points for each individual. We fit two models: 1) to evaluate the effects of the HDT and HDT + AG environment on performance and 2) to evaluate recovery after exiting the HDT and HDT + AG environment. These behavioral data were analyzed and presented in a previous published work (Tays et al., 2022); the data are included here for brain-behavior correlation analyses.

### 2.7 Subject-Level fMRI Statistics

For each individual subject, and at each time point, we calculated brain activation, and deactivation, on a voxel-by-voxel basis for vestibular stimulation versus rest. As in our previous longitudinal fMRI work, we set the first level masking threshold to –infinity (Hupfeld et al., 2020; 2021; Salazar et al., 2020; 2021; Tays et al., 2023.

### 2.8 Group Level Statistical Analyses

Before testing for any group differences, we first verified that our pneumatic tapper was elicited the expected results. Next, to determine whether AG mitigated the effects of HDT we used multiple statistical models. Here, we assessed longitudinal changes comparing the groups in specific regions of interest (ROI) that showed changes in our prior HDT and spaceflight studies for activity in response to vestibular stimulation (described in 2.8.1). Then, we tested hypothesis-driven longitudinal changes comparing subjects that received the centrifugal AG daily and the controls (described below in 2.8.2). Finally, we tested for brain-behavior correlations to evaluate whether participants’ activation changes across HDT were related to their balance and mobility performance changes.

#### 2.8.1. Main Effect Analysis

In order to verify the pneumatic tapper method was working, we first tested the main effect of vestibular stimulation across all participants prior to entering the HDT environment at BDC-7. We set our statistical threshold at FWE < 0.05 while controlling for age and sex.

#### 2.8.2. ROI Brain-Behavior Correlation Analysis

We performed brain-behavior correlations to examine the association between any brain changes and changes to mobility and balance occurring with HDT and AG. Here, we assessed whether changes in brain activity from BDC-7 to HDT58 were related to changes in mobility and balance behavior from BDC-1 to R + 0 within our 10 pre-designated ROIs. We assessed this through Pearson correlation analysis conducted within R 3.6.1. (R Core Team, 2022).

#### 2.8.3. Exploratory Time Course of Neural Response to HDT + AG

To test the potentially mitigating effect of AG on HDT-induced changes, we implemented an *a priori* hypothesized weighted longitudinal model in a whole brain, exploratory manner. We created longitudinal contrasts that include pre-HDT time point BDC-7, HDT29 and HDT58 to investigate vestibular processing changes directly due to HDT. We also assessed recovery in these regions by assessing functional brain changes from HDT58 to R + 10, but only examining regions that changed due to HDT. The models were built using the Sandwich Estimator Toolbox for SPM12 (SwE; Guillaume et al., 2014), similar to as we have done in previous spaceflight analog investigations (Yuan et al., 2016; 2018a; 2018b; Hupfeld et al., 2020; 2021, Salazar et al., 2020; 2021; McGregor et al., 2021). The SWE toolbox uses a noniterative marginal model to prevent within-subject convergence problems inherent to longitudinal designs, providing optimal analysis of longitudinal MRI data, especially with small data sets and missing data. The SwE default setup was used, modified only to use non-parametric wild bootstrapping with 999 permutations, which is recommended for small sample sizes (Guillaume & Nichols, 2015). Mean centered age and sex were included in the model as covariates. Significance was analyzed at a *p* < 0.05, family-wise error (FWE) corrected for multiple comparisons. For whole brain analysis, an explicit mask was used to investigate only gray matter effects in the cerebrum (and not the cerebellum, which was analyzed separately). This mask was created through binarizing the Computational Anatomy Toolbox 12 (CAT12; Dahnke et al., 2013; Gaser & Kurth, 2017) MNI-space gray matter template at a threshold of 0.1. Recovery was assessed only in regions that showed changes due to HDT by creating a results mask and implementing it with the recovery model. Cerebellar analyses were conducted only on the cerebellum as discussed above in “Cerebellar Pre-processing.”

## 3. Results

We initially examined for statistical differences in brain activation between the two AG groups (continuous and intermittent) and found none; thus we pooled them together into a joint AG group.

### 3.1 Main Effect

Like our and others’ previous work (zu Eulenburg et al., 2012; Lopez et al., 2012; Noohi et al., 2017; 2019; Yuan et al., 2018; Hupfeld et al., 2019; 2022), vestibular stimulation versus rest resulted in activation in the right and left rolandic operculum, right temporal supplementary region, right hippocampus, right para-hippocampus and cerebellar lobule 8 at the baseline condition (prior to entering HDT). Further, we observed deactivation in widespread somatosensory, motor and occipital regions (Supplementary Fig. 1) as we expected. This analysis was conducted with all subjects in one group, prior to entering HDT all participants are treated similarly and naïve to the group designation.

### 3.2 ROI Based Analyses

In our ROI analyses, we identified a significant group by time interaction in the right cerebellar lobule VI (Table 1; Fig. 4). That is, CTRL subjects on average showed a decrease in cerebellar activation during vestibular stimulation, whereas the AG group showed no change from pre- to late-HDT. Further, we identified group differences in the L superior frontal, R pre-central gyrus and L OP2 ROIs, where the AG group had less deactivation throughout all time points, including pre-HDT.

### 3.3 ROI Brain-Behavior Correlations

The functional mobility and balance data are more fully presented in a separate publication (Tays et al., 2022); here we use the same behavioral data with the ROI based analysis data in a brain-behavior change-change correlation analyses. We identified a significant change-change correlation in the AG group’s L OP2 activation and their SOT-5 performance (*p* = 0.019, t = 2.6911). Those that showed greater decreases in activation in this region at later HDT, compared to pre-HDT, showed less pre to post HDT balance declines (SOT-5 measure).

### 3.4 Exploratory Whole Brain HDT + AG

**Figure 3:**
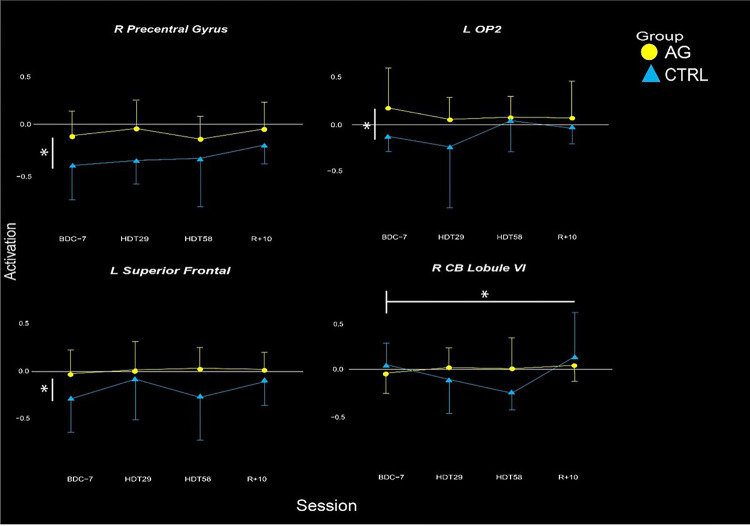
Activation patterns in the four ROIs that showed significant changes or differences reflected in Table 3. The AG group is colored in yellow, whereas the CTRL group is blue.

We identified two clusters in the cerebral cortex that significantly differed in their activation profiles from pre- to post-HDT between groups (*p*_FWE–corr_<0.05, cluster size k > 5; Table 2). In these clusters, we identified that the AG group showed no statistical changes in activation after entering the HDT environment. However, the CTRL group decreased deactivation in both regions after entering HDT. In the precentral gyrus, the CTRL group showed initial increases in activation that continued to increase until HDT-58, where it then reached a plateau. In the inferior frontal gyrus, the CTRL participants had a large initial increase in activation that remained elevated throughout HDT and even through the recovery phase (Fig. 3). In both of these regions, the AG group showed relatively stable levels of activation.

## 4. Discussion

**Figure 4:**
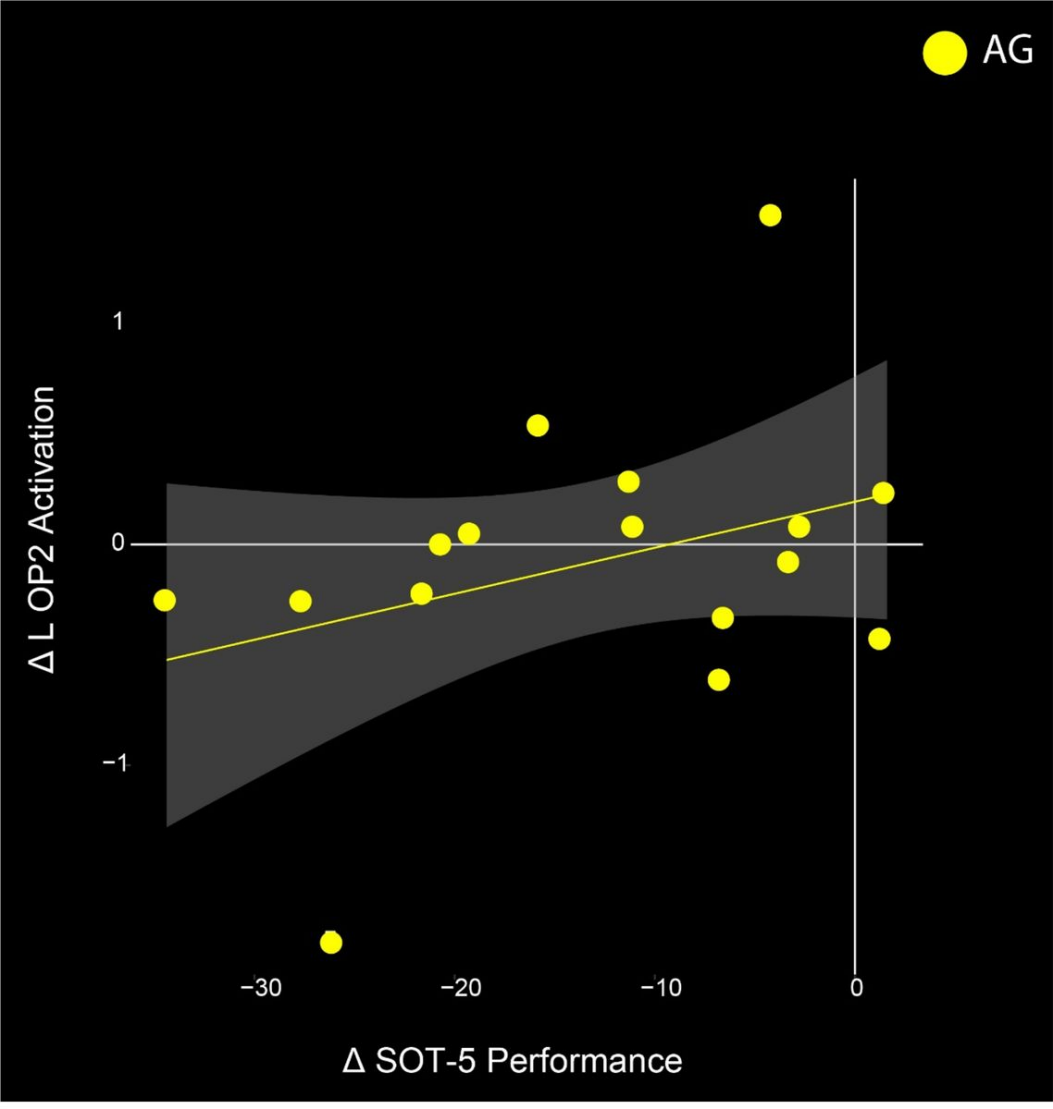
Brain and Behavior correlation. Change in the L OP2 ROI significantly correlates with change in the SOT-5 task performance from pre-to late-HDT in the AG group. The two most extreme data points do not qualify as statistical outliers.

**Figure 5:**
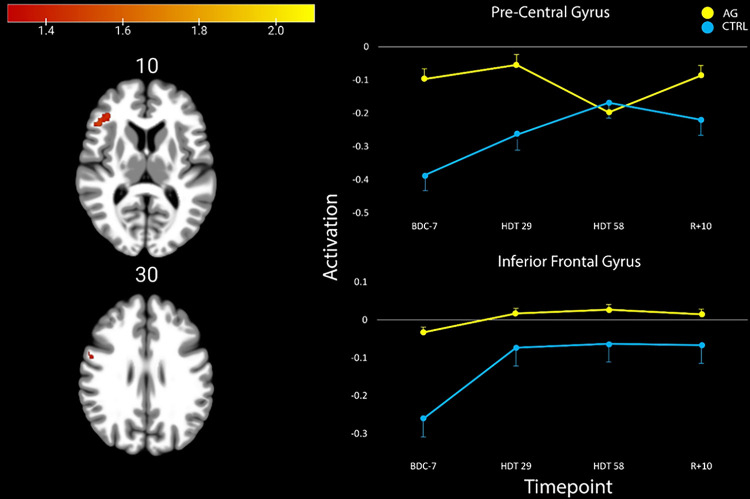
Group differences in activation after entering the HDBR environment. The AG group showed an overall lack of change in HDBR, whereas the CTRL group showed increased activation in both the pre-central gyrus and the inferior frontal gyrus.

This study investigated the use of AG to serve as an integrated countermeasure to mitigate HDT induced declines in vestibular processing and balance. We identified two clusters of brain activity exhibiting group (AG vs CTRL) by time interactions, in the precentral gyrus and the inferior frontal gyrus (Fig. 3, Table 2). We also investigated ten ROIs that are active in response to vestibular stimulation and that exhibited changes in other HDT studies. Within these ROIs we identified three group main effects, and a group by time interaction in the right cerebellar lobule VI. In cerebellar lobule VI, activity remained stable throughout HDT for the AG group whereas the CTRL group decreased cerebellar activity during HDT and then recovered towards baseline levels post-HDT (Fig. 4, Table 3). Further, we found that those in the AG group that maintained their activation in the left OP2 region the most when entering HDT had the smallest decreases in balance from pre- to post-HDT (measured on the SOT-5 test, Fig. 5). Overall, these findings suggest that AG can mitigate some of the vestibular processing effects typically associated with HDT. We suggest that AG should be further explored as an integrated countermeasure.

To assess our primary hypothesis we performed ROI analyses, extracting activation patterns from ten ROIs that we have shown in prior studies to be activated in response to vestibular stimulation and to change with HDT. Of the ten ROIs, one in the right cerebellar lobule VI showed a significant group by time interaction. In this ROI, the AG group’s activation remained stable after they entered the HDT environment, but the CTRL group displayed decreasing deactivation after entering HDT. CTRL activation returned to baseline levels following the exit of the HDT period (Fig. 4). Lobule VI has previously been shown to be active during performance of complex sensorimotor tasks, and it shows topographical differences in hand versus foot movements (Schlerf et al., 2010). Further, this region has also been shown to be engaged during n-back working memory tasks in conjunction with lobule VII (Grodd et al., 2005). Lobule VI has also been shown to atrophy in patients that have cerebellar ataxia, neuropathy and vestibular areflexia (CANVAS), along with lobules VIIa and VIIb (Rust et al., 2017; Infante et al. 2018), supporting that it plays a role in vestibular processing and balance. Anatomical lesions in this lobule, as well as in lobules V and VIIa, have also been shown to hinder vestibular compensation in patients that have had a cerebellar stroke (Baier et al., 2015). Thus we speculate that the use of AG in this HDT campaign affects this region as part of a complex network that receives vestibular and somatosensory inputs and integrates them to perform vestibular mediated sensorimotor tasks.

To examine the potential functional consequences of the observed brain changes, we conducted a brain-behavior change-change correlation analysis with the 10 ROIs and mobility and balance measures collected during this campaign. In the AG group, we identified a significant correlation between pre- to late-HDT activation change in the left OP2 region and the change from pre- to post-HDT SOT-5 balance condition. Participants that received AG and maintained their pre-HDT levels of activation in this region showed the least balance declines after exiting HDT (Fig. 4). The OP2 region has been suggested to be the human vestibular cortex (zu Eulenburg et al., 2012; Ibitoye et al., 2023). Importantly, the SOT-5 balance condition specifically engages vestibular processing. The visual system is perturbed (eyes closed) and proprioception is unreliable (sway referenced platform), driving an increased reliance upon the vestibular system. The findings here indicate that those that received AG and subsequently were able to maintain their pre-HDT levels of activation were also able to preserve their performance in this balance condition the most. This suggests that AG may be a successful counter-measure for balance in this spaceflight analog, however there are individual differences in the extent of its effectiveness.

To identify if additional regions outside of our pre-selected ROIs showed changes due to HDT and AG, we conducted an exploratory whole brain analysis. We found that after entering bedrest, the CTRL group increased activation in both the pre-central gyrus and the inferior frontal gyrus when receiving vestibular stimulation. In the pre-central gyrus activation continued to increase until HDT-58, whereas the AG group had no changes upon entering HDBR, and slightly decreased activation by HDT-58. Following HDT, the CTRL group decreased activation towards their pre-HDT levels and the AG returned to their baseline activation levels. In the inferior frontal gyrus region, the AG group on average showed no change, while the CTRL group maintained their increased activation inside of HDT and during the recovery period. The precentral gyrus plays a key role in voluntary movement execution (Banker & Tadi, 2019; Chouinard & Paus, 2006). We have previously identified that this region is activated via vestibular stimulation (Noohi et al., 2017), and shows a steeper slope of activation change during 70 days of bedrest compared to non-HDT controls (Yuan et al., 2017). Moreover, we recently reported increased activation in the precentral gyrus in response to vestibular stimulation following long duration spaceflight (Hupfeld et al., 2022). This is comparable to what we observed here in the CTRL group, whereas AG mitigated the effect. This supports that AG effectively mitigates changes in brain vestibular activity that occur with HDT; due to the similar changes that occur with spaceflight, this finding suggests that AG may be an effective countermeasure to spaceflight induced changes as well.

In addition to the precentral gyrus, we identified increased activation within the right inferior frontal gyrus (rIFG). CTRLs showed an increase in activation upon entering HDT that persisted throughout the 60 days, and the following 10 days of recovery (Fig. 5). The rIFG region has been repeatedly shown to be connected to a variety of functions, including sensorimotor, but has a considerable role in response inhibition and attentional control (Hampshire et al., 2010). Interestingly, this region is not frequently associated with vestibular function, however in patients with acute vestibular neuritis there has been an identified association where an increased degree of nystagmus is associated with increased regional cerebral glucose metabolism in the rIFG (Bense et al., 2004). Further, recall of vestibular sensation has been shown to activate both the rIFG and the left IFG (zu Eulenburg et al., 2012), however the left IFG is more typically associated with vestibular function. It is possible that activation of the rIFG in this instance is indicative of compensatory recruitment of the contralateral IFG. However, the rIFG is also reliably shown to be associated with motor inhibition through a variety of lesion and brain stimulation studies (Aron et al., 2007; Ditye et al., 2012; Chambers et al., 2006; Stramaccia et al., 2015). Motor inhibition is a vital aspect of conducting human movement as it allows a person to suppress inappropriate actions (preventing oneself from a harmful action), interrupting current actions (releasing the gas pedal to brake) and plays a key role in movement disorders (Duque et al., 2017; Ganos et al., 2018). Increased activation within this region in the control subjects and specifically associated with HDT could suggest a negative effect or increased cost of HDT on motor inhibition, which in this context, could lead to increased risk form these environments.

During the AGBRESA campaign, NASA, ESA and the DLR investigated whether AG could serve as an integrated countermeasure to target physiological and neurological deficits induced by HDT that models spaceflight. The overall findings of this campaign have varied widely, but within the sensorimotor domains there have been promising findings. Multiple investigations have identified AG to have some positive effect on balance and vestibular function (De Martino et al., 2021; Tays et al., 2022). We have also identified that it may increase neural efficiency in sensorimotor adaptation tasks (Tays et al., 2023). We found that participants performed the paced serial audition test better during centrifugation (Tays et al., 2022), but daily AG seems insufficient to counteract HDT-induced cognitive declines (Basner et al., 2021). It is possible that AG primarily works on sensorimotor and vestibular functions, as we see here that it has some effects on specific brain regions and function. However, further investigation is needed to understand its mechanistic effects and individual differences in responsivity.

### Limitations

The study here has several limitations that should be considered when interpreting the findings. First, participants were randomly split between the three groups, however, in three of our ten ROIs main effects of group were identified, suggesting that even prior to HDT there may have been some differences between the groups. Second, the sample size is limited, making it more difficult to identify subtle group and individual differences. Future investigations should increase the sample size to increase power and interpretability. Third, the current HDT campaign was only 60 days, whereas ISS missions typically take around 180 days. This may result in only partial dysfunction in HDT compared to astronauts. Lastly, AG may require individualized dosing. Here, the rotational speed was calculated to create a similar level of G in the z axis at the CoM, however the amount of time each participant received this was standardized to 30 minutes.

Here, we investigated the use of AG as an integrated countermeasure to target vestibular brain changes that occur with HDT, a spaceflight analog. The AG group received 30 minutes per day for 60 days, resulting in no activation changes in a cerebellar ROI, as well as the right precentral gyrus and right IFG in an exploratory whole brain analysis. In contrast, the CTRL group showed activation changes specific to HDT in these regions. We also identified a brain and behavior change-change correlation in the left OP2 ROI, where those that received AG and maintained their pre-HDT levels of activation in this region showed the least balance declines from pre- to post-HDT. We interpret these findings to suggest that AG may increase sensory stimulation and could result in preserving vestibular system function, even in environments where this system’s function typically declines. These findings support that further investigation into AG as a HDT and spaceflight countermeasure should be conducted.

## Figures and Tables

**Figure 2:**
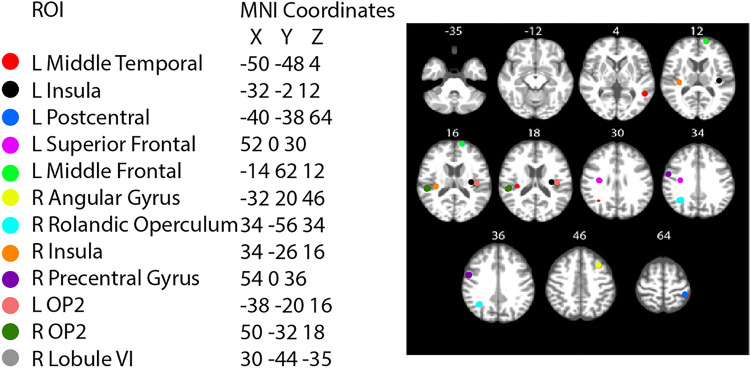
ROI spheres placement.

**Table 1:** ROI based analysis

	HDT + AG		Group		HDT		Age		Sex	
ROI	β	*P*	B	*p*	β	*p*	β	*p*	β	*p*
L Middle Temporal	−0.000	0.896	−0.053	0.680	0.003	0.143	−0.004	0.504	0.063	0.896
L Insula	0.001	0.532	−0.156	0.079	−0.001	0.632	−0.000	.930	0.046	0.524
L Postcentral	−0.012	0.199	−0.232	0.543	0.000	0.935	−0.138	0.378	−0.412	0.200
L Superior Frontal	−0.001	0.842	−0.280	**0.042**	0.001	0.641	0.001	0.843	−0.052	0.630
L Middle Frontal	−0.005	0.143	−0.013	0.923	0.001	0.773	0.001	0.846	0.046	0.687
R Angular Gyrus	−0.001	0.670	−0.021	0.857	0.001	0.731	−0.002	0.701	−0.012	0.905
R Rolandic Operculum	0.001	0.593	−0.238	0.108	−0.003	0.427	0.005	0.504	−0.050	0.713
R Insula	0.002	0.670	−0.267	0.143	−0.002	0.300	−0.014	0.080	−0.009	0.952
R Precentral Gyrus	0.002	0.468	−0.294	**0.044**	−0.001	0.691	0.005	0.468	−0.115	0.277
L OP2	0.005	0.191	−0.342	**0.046**	−0.002	0.408	−0.001	0.881	−0.076	0.571
R OP2	0.006	0.183	−0.109	0.650	−0.003	0.329	−0.003	0.818	−0.201	0.368
R Lobule VI	−0.005	**0.037**	0.067	0.518	0.001	0.647	−0.005	0.229	−0.099	0.247

Note

Values that are significant are bolded under the effect. ROI is the region of interest that is being assessed in their respective column. HDT + AG refers to the group by time interaction of artificial gravity impacting the effects of HDT. HDT refers to the effects of time spent in the HDT environment. Beta values are listed under **β** to inform direction and strength of the relationship, while statistical values are listed under **p**. R and L are Right and Left, respectively.

**Table 2:** Regions that exhibited group by time effects.

	TFCE Level		MNI Coordinates (mm)		
	*P*_FWE-corr_	Extent (k_E_)	*X*	*Y*	*Z*
Inferior Frontal Gyrus, R	0.032	235	48	26	10
Precentral Gyrus, R	0.041	50	54	2	30

Note: Significance set at *p*_FWE-corr_<.05, FWE corrected and k > 5. Clusters were labelled based on the AAL atlas. AG = Artificial Gravity. HDT = Head Down Tilt. R = Right.
